# The Surgical Aid Society

**Published:** 1893-12-09

**Authors:** 


### THE SURGICAL AID SOCIETY.

There was an unusually large attendance—which included a number of ladies—at the annual meeting of this Society, held in Cannon Street Hotel on Monday afternoon. The Lord Mayor presided. Apologies were intimated from Mr. Alderman and Sheriff Moore, Sir J. Whitehead, M.P., Sir Whittaker Ellis, Alderman Dimsdale, Mr. C. E. Tritton, and others. A very gratifying feature of the annual report, read by Mr. Tressider, the secretary, was that, notwithstanding bad times, the revenue of the Surgical Aid Society has been well maintained. The sum derived from annual subscriptions during the past twelve months is again in advance of the previous year, having reached considerably over £6,000. The annual revenue from all sources amounts to over £10,000, which, as Mr. Tressider remarked, compares favourably with £136, which was the Society's income for the first year of its existence. The increasing number of contributions from the employés of large firms and associations of working men is noted with satisfaction. In order to meet current expenses it has been necessary to borrow £500 from the bankers; it is hoped that this advance will speedily be repaid; but it was pointed out, as the Society has over £10,000 invested, it cannot be correctly said that in obtaining this temporary loan it has gone into debt. The report gives details as to the work that has been done during the year. Ten thousand nine hundred and ten patients have been relieved, an average of 210 per week; in all, 17,560 appliances have been issued to the poor—the average cost of each appliance was 12s. 0½d. The Society has been extending its operations throughout the country. New branches have been formed at Brighton, Leicester, and Manchester. Croydon, which is the oldest branch, is still to the front with an income of £207. The department of the Society's work in connection with which loans have been made to the poor of air and water beds, invalid chairs and carriages, and other appliances, has proved the means of alleviating much suffering, and in not a few instances of enabling the maimed and halt to earn a living. "The committee rejoice in the present solid prosperity of the Society, and hopefully contemplate a still larger and more useful future." Many speakers at the meeting testified to the good work done by the Society, in which the Lord Mayor was invited to fill the office of vice-president, which he cordially accepted.

